# LA-ICP-MS protocols for U-Pb geochronology and trace element analysis of zircons: Optimized implementation at the PetroChron laboratory^1^

**DOI:** 10.1016/j.mex.2026.104014

**Published:** 2026-06-23

**Authors:** Maria Pârlea, Vlad Ene, Mihai Ducea, Peter Luffi, Ciprian Stremțan

**Affiliations:** a Doctoral School of Geology, Faculty of Geology and Geophysics, University of Bucharest, Romania; b Faculty of Geology and Geophysics, University of Bucharest, Bucharest, 010041, Romania; c Geological Institute of Romania, Bucharest, Romania; d Sabba Ștefănescu Institute of Geodynamics, Romanian Academy, Bucharest, Romania; e Teledyne Photon Machines, 512 East Madison Ave, Belgrade, MT, 59714, USA

**Keywords:** Zircon geochronology, Zircon geochemistry, U-Pb dating, LA-ICP-MS optimization

## Abstract

This article presents a standardized analytical protocol for zircon characterization using a 193 nm ArF excimer laser (Teledyne Photon Machines IRIDIA) coupled to a single quadrupole ICP-MS (Thermo Scientific iCAP RQ). The method optimizes two primary workflows: high-resolution U-Pb geochronology and the simultaneous acquisition of U-Pb isotopic ratios with trace element (TE) concentrations. By systematically calibrating operational parameters—including carrier gas flow, repetition rates, and laser fluence—this approach maximizes analytical sensitivity while minimizing sample consumption, providing a robust framework for investigating detrital, igneous, and metamorphic zircons.

• Optimized Workflows: Establishes precise parameters for both standalone U-Pb dating and integrated U-Pb/Trace Element analysis to ensure high spatial resolution and analytical throughput.
• Performance Metrics: Demonstrates U-Pb age accuracy within 5% (precision <3%) and trace element accuracy better than 10% for most analytes based on extensive data acquisition.
• Petrogenetic Application: Delivers a reliable, integrated framework for complex geological characterization, maintaining high-standard precision across diverse zircon populations.

## Graphical abstract

**Figure:**
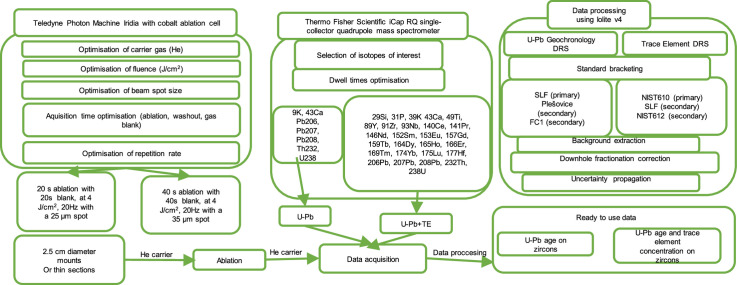

**Specifications table**

**Table:** 

**Subject area**	Earth and Planetary Sciences
**More specific subject area**	U-Pb and trace elements analysis on zircon
**Name of your method**	LA-ICP-MS analysis on zircons
**Name and reference of original method**	None
**Resource availability**	None

## Background

Zircon (ZrSiO4) is widely regarded as the premier geochronometer in Earth Sciences due to its unique crystallographic and chemical properties [1]. During crystallization, the zircon lattice effectively excludes common lead (^204^Pb) due to charge and ionic radius incompatibilities, while substituting significant concentrations of uranium (U) and thorium (Th) [2]. Consequently, virtually all measurable radiogenic lead (^206^Pb, ^207^Pb, ^208^Pb) within the crystal is the product of *in-situ* radioactive decay, facilitating high-precision age determinations [3]. Furthermore, zircon exhibits exceptional mechanical and chemical stability, maintaining an isotopically closed system even under extreme crustal conditions, including high-grade metamorphism and physical transport. Beyond geochronology, zircon's ability to incorporate trace elements (e.g., Hf, Ti, Y, REE) provides a valuable geochemical fingerprint of the igneous or metamorphic process driving its growth. Because zircon is chemically and mechanically exceptionally resistant, it commonly maintains a "closed-system" behaviour. In modern petrology, U-Pb dating of zircon provides critical temporal constraints on diverse geological phenomena, including the crystallization of magmatic suites, the timing of metamorphic overprints, and the characterization of sediment provenance through detrital zircon populations [4,5].

Laser ablation- inductively coupled plasma-mass spectrometry is a powerful analytical technique that combines laser ablation with mass spectrometry to spatially resolve the elemental composition and isotopic ratios in solid samples. This technique has been used for >30 years and offers high spatial resolution and sensitivity, making it ideal for precise geochronological studies [[6], [7], [8]].

The precision and accuracy of U-Pb geochronology and for trace elements concentrations are directly influenced by the parameters chosen for data acquisition and analysis [9]. Parameters encompass a wide range of factors, including instrumental settings, data processing algorithms, and criteria for data selection and rejection [3]. Each parameter set can significantly impact the resulting measurement errors. These uncertainties characterize the confidence intervals associated with the calculated ages, critically influencing the interpretations of geological processes and timelines [10]. Researchers continually refine these parameters to achieve the highest precision and reliability in age determinations [11] and in chemical composition of the analysed mineral. However, the optimal parameter set may vary depending on factors such as the type of sample (e.g., zircon, monazite), the presence of common Pb, the chemical variations within a single crystal, the geological context etc., [2].

The PetroChron Laboratory (University of Bucharest) is the only laboratory in Romania that has the capabilities to measure isotopes using LA-ICP-MS. Analytical protocols employed here are optimized for two primary workflows: Method 1: U-Pb dating on zircons (U-Pb) and Method 2: Trace elements measured simultaneously with U-Pb isotope ratios on zircons (U-Pb+TE). While from a data quality perspective analysing U-Pb isotope ratios and trace elements separately is in general preferable, the latter method may often offer a better solution for acquiring coupled geochronology-geochemistry (i.e.*,* petrochronology) results in the case of small or highly heterogeneous grains.

## Method details

The system consists of a Teledyne Photo Machines IRIDIA 193 nm ArF excimer laser ablation system coupled to a Thermo Fisher Scientific iCAP RQ single quadrupole mass spectrometer. High-purity gases are utilized to ensure signal stability and component longevity: high-purity He 6.0 serves as the carrier gas and Ar 5.0 sustains the ionizing plasma (Table 1). Pre-analytical calibrations include a cell evacuation protocol prior to each session to minimize atmospheric contamination and a 30-minute plasma stabilization period while monitoring 26 masses between ^7^Li and ^238^U in gas blank.

**Table 1:** PetroChron laboratory specifications.

Laboratory and sample preparation	
Laboratory name	PetroChron Laboratory at University of Bucharest
Sample type/ mineral	Zircon
Sample preparation	Standard mineral separation, 2.5 cm resin mount, 5 μm polish
**Laser ablation system**	
Make, model and type Ablation cell and volume	Teledyne Photon Machine IRIDIA with Cobalt two-volume ablation cell
Laser wavelength	193nm
Energy density/Fluence	4 J/cm^2^
Repetition rate	20 Hz
Spot size and pattern	25–35 μm circular spot
Carrier gas and flow	Helium 0.3 L/min (inner cell) 0.3 L/min (outer cell)
Ablation duration	20–40 s
**ICP-MS instrument**	
Make, model and type Sample introduction	Thermo Fisher Scientific iCAP RQ single-collector quadrupole mass spectrometer
RF power	1350 W
Make-up gas flow	0.95 L/min Ar (gas mixed with He carrier gas inside glass cyclonic spray chamber)
Detection system	single detector
Measured masses and their integration time per peak (mass)	^29^Si=0.001 s; ^141^Pr=0.25 s; ^169^Tm=0.01 s; ^31^P = 0.005 s; ^146^Nd=0.2 s; ^174^Yb=0.005 s; ^39^K =0.001 s; ^152^Sm=0.15 s; ^175^Lu=0.005 s; ^43^Ca=0.001 s; ^153^Eu=0.04 s; ^177^Hf=0.001 s; ^49^Ti=0.01 s; ^157^Gd=0.05 s; ^206^Pb=0.0192 s; ^89^Y=0.001 s; ^159^Tb=0.03 s; ^207^Pb=0.015 s; ^91^Zr=0.001 s; ^164^Dy=0.03 s; ^208^Pb=0.0657 s; ^93^Nb=0.035 s; ^165^Ho=0.01 s; ^232^Th=0.0017 s; ^140^Ce=0.01 s; ^166^Er=0.01 s; ^238^U=0.0012 s;
Integration time per reading	0.97s
**Measurement procedures**	
Gas blank	20–40 s
Calibration strategy	U-Pb ages: SLF zircon (calibration material), FC1 and Plešovice zircons (secondary standards) TE: NIST610SRM glass (calibration material), SLF zircon and NIST612SRM glass (secondary standards)
Reference materials	SLF (563.2 ± 16 Ma; [5]) Plešovice (337.1 ± 0.2 Ma; [12]) FC1 (1099.5 ± 0.5 Ma; [13]) NIST610SRM [14] NIST612SRM [14]
**Data processing**	
Data processing package	Iolite v4
Mass discrimination	^207^Pb/^206^Pb and ^206^Pb/^238^U are normalised to primary reference material (SLF Zircon) analysed repeatedly throughout the analytical session. The results are validated by regular analysis of secondary reference zircons of known age (FC-1, Plešovice).
Uncertainty level and propagation	Ages are quoted at 2 *s* absolute uncertainty. Uncertainty is propagated by quadratic addition of excess variance, the primary component of which is based on the reproducibility of the primary reference material (SLF and NIST610SRM).

The two methods employed in the PetroChron Lab (U-Pb and U-Pb+TE) are routinely performed on 2.5 cm diameter epoxy mounts or in thin sections (for U-Pb), provided enough zircon grains exist. All types of zircons are analysed, from igneous and metamorphic to detrital zircons. The process for unknown samples starts with separation procedures.

The preparation process for epoxy-mounted zircons involves several stages of mechanical processing and physical separation. Initially, rocks are crushed into 2–5 cm chips using a Retsch BB200 jaw crusher, followed by further grinding to approximately 350 μm in a Retsch DM200 disc mill. The ground material is then processed on a Wilfley table to remove low-density minerals like quartz and plagioclase through hydrodynamic separation. Following this, a Frantz isodynamic separator is used to sort minerals by magnetic susceptibility. This is performed in successive stages by increasing the electromagnet current from 0.4 A at a 20° incline up to 1.2 A at a 5° incline. The remaining non-magnetic fraction then undergoes heavy liquid separation using a sodium polytungstate (SPT) solution with a density of up to 3 g/cm³. In the final stage, zircon grains are either manually picked (igneous and metamorphic samples) from the heavy fraction under a binocular microscope and mounted or directly mounted en-mass (detrital samples) in epoxy pucks for analysis.

The setup for the laser ablation includes some parameters that are set similarly for both U-Pb and U-Pb+TE methodologies and other parameters that are set differently for the two methodologies. The parameters that remain constant across methodologies include the flow rate of the carrier gas (He). Experiments aimed at balancing detection limits against background noise indicate that an optimal signal-to-noise ratio is achieved at a He flow of 0.3 L/min in both the inner and outer cells. An increased flow rate elevates background noise, potentially obscuring low-concentration isotopes. Conversely, an insufficient flow rate results in inadequate material transport, preventing low-concentration isotopes from being distinguished from the background; laser repetition rate is maintained at 20 Hz for both methodologies, as it represents the optimal balance between the speed of analysis and the downhole fractionation during single-spot mineral analysis. A lower repetition rate increases the time needed for an analysis, whereas a higher repetition rate risks to produce the piercing of the zircon grains prematurely. Laser fluence (power density) was also evaluated. While initial measurements utilized 3.6 J/cm^2^, subsequent increases up to 4.0 J/cm^2^ demonstrated that zircon measurement outcomes had increased signal.

Regarding the parameters that are different for the two methodologies, these include: beam spot size, which is 25 μm for U-Pb, which allows for the analysis of smaller grains without compromising precision, while 35 μm is the minimum spot size for U-Pb+TE, allowing for enough material to be ablated for the simultaneous detection of 27 isotopes; acquisition times that include ablation, washout and gas blank are similarly scaled based on the analytical requirements. While a 20-second ablation time with a 20-second background (2 s washout, 18 s gas blank) is sufficient for the short isotope list used in U-Pb method, U-Pb+TE method requires a 40-second ablation period. This is accompanied by a 40-second gas blank period, consisting of 10 s for washout and 30 s for background.

To obtain statistically substantiated zircon U-Pb ages, a minimum of 30 measurements are required in the case of igneous and metamorphic samples, whereas in the case of detrital samples the analysis of at least 120 zircons is necessary [15]. Likewise, at least 30 analyses are required to characterize trace element concentrations in igneous and metamorphic zircons.

The list of isotopes analysed for U-Pb method include ^206^ Pb, ^207^Pb, ^208^Pb, ^232^Th, ^238^U for dating and ^31^P, ^39^K, ^43^Ca for contamination detection. ^204^Pb, and ^235^U are not routinely analysed. ^204^Pb is redundant as mentioned above, and would be hard to measure due to interferences with ^204^Hg which can be found in trace amount in the carrier gas, and ^235^U is calculated by Iolite using the constant ratio ^238^U/^235^U= 137.8180 [16].

The U-Pb + TE list includes the following isotopes: ^29^Si, ^31^P, ^39^K, ^43^Ca, ^49^Ti, ^89^Y, ^91^Zr, ^93^Nb, ^140^Ce, ^141^Pr, ^146^Nd, ^152^Sm, ^153^Eu, ^157^Gd, ^159^Tb, ^164^Dy, ^165^Ho, ^166^Er, ^169^Tm, ^174^Yb, ^175^Lu, ^177^Hf, ^206^Pb, ^207^Pb, ^208^Pb, ^232^Th and ^238^U The dwell times for each element was calculated with the help of HDIP modelling program (Teledyne Photon Machines, software version:1.9.1), taking into consideration their relative abundances in zircons. Their dwell times can be found in Table 1.

Data reduction was conducted in Iolite 4 [17]. U-Pb ages were processed using the *U-Pb Geochronology* data reduction scheme (DRS) [18], while trace elements were processed via the *Trace Element* DRS [19]. The internal standard used for zircons was Si, which was measured stoichiometrically.

Instrumental drift and down-hole fractionation were corrected using a strict standard-sample bracketing sequence: two primary standards were measured at the beginning and end of each run, with one primary standard inserted after every five unknown samples. Secondary standards were distributed throughout the session to monitor instrumental drift, particularly because background signals decrease over the course of an analytical day. To ensure statistical robustness, the external reproducibility of the primary standards was propagated in quadrature to the internal errors of the unknown samples. Final ages and elemental concentrations are reported at the 2 s uncertainty level. Data discrimination after the DRS was done via visual inspection of the time-resolved signal of ^31^P,^39^K and ^43^Ca which helped eliminate data points that showed chemical evidence of mineral inclusion (such as apatite, silicate melt inclusions etc.) and other imperfections in the analysed zircons (such as cracks, fractures etc.) and filtering for U-Pb age discordances >10% in the case of age dating.

Analytical protocols varied depending on the target analytes. For U-Pb geochronology in zircon, SLF (563.2 ± 16 Ma; [5]) served as the primary reference material. Accuracy was monitored using secondary standards including FC1 (1099.5 ± 0.5 Ma; [13]), Plešovice (337.1 ± 0.2 Ma; [12]), and occasionally R33 (419.3 ± 0.4 Ma; [20]).

For TE analysis, NIST610SRM [14] (synthetic glass) served as the primary standard, while NIST612SRM [14] and most importantly, SLF were used as secondary standards. To address potential matrix-induced fractionation between the synthetic glass primary standard and the unknown samples, the SLF zircon (values determined by [21]) was utilized as a critical matrix-matched monitor. SLF is well characterised in Y and REE composition with a lower variation, compared with other natural zircon standards. 91,500 [22,23], should be used as primary standard in this kind of methodology, but due to is scarcity, it was used for validation in a limited number of runs (6 runs) only. For the U-Pb+TE methodology, a dual-standard calibration was employed, in the standard run NIST610SRM was used as primary standard for chemistry, and the samples are bracketed a second time with SLF, which is used as secondary standard for chemical composition and as a primary for geochronology.

## Method validation

Routine protocols were developed between 2023 and 2024, with validation data compiled from 26 U-Pb sessions and 16 U-Pb+TE sessions conducted between June 2025 and March 2026. For each session, weighted means and propagated errors were calculated. Sample sizes varied by session. The number of valid Plešovice zircons analyses was n = 450 (U-Pb) and n = 160 (U-Pb + TE); in the case of FC1 zircons, n = 292 (U-Pb), and n = 152 for U-Pb+TE.

**Fig. 1:**
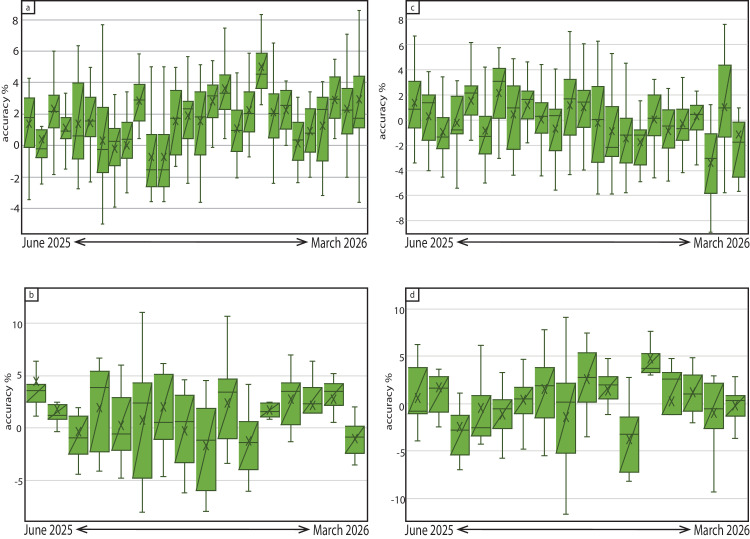
Box-and-whiskers plots representing age accuracies from each session shown as the differences between measured and accepted values, in %. The horizontal line represents the average and the x represents the median. (a) U-Pb sessions for Plešovice; (b) U-Pb+TE sessions for Plešovice; (c) U-Pb sessions for FC1; (d) U-Pb+TE sessions for FC1.

From a geochronological perspective, both U-Pb and U-Pb+TE methods yielded ages with accuracies of 5% ±1% for both Plešovice and FC1. The most noticeable difference between sessions is the spread of data. All accuracies obtained with the U-Pb method are within 5% in the case of Plešovice (Fig. 1a) and within 6% in the case of FC1 (Fig. 1c), the median accuracy being ∼2% for both standards. In contrast, results for the U-Pb+TE method show a wider spread. For the majority of the sessions, the accuracy is within 5% (Plešovice) (Fig. 1.b) and 6% (FC1) (Fig. 1d), the median accuracy staying within 2% (Plešovice) and 3% (FC1).

**Fig. 2:**
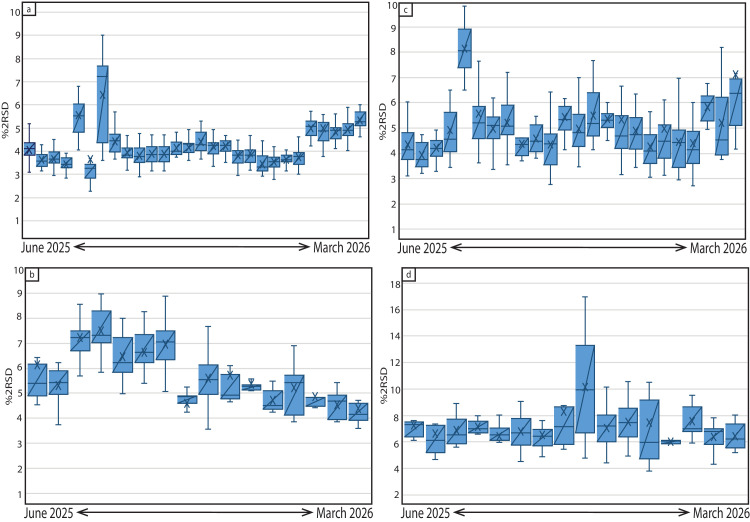
Box and whiskers plots representing variation in 2 s precision measured in %2RSD. The horizontal line represents the average and the x represents the median. (a) U-Pb sessions for Plešovice; (b) U-Pb+TE sessions for Plešovice; (c) U-Pb sessions for FC1; (d) U-Pb+TE sessions for FC1.

In the case of the 2 s precisions, the difference between the U-Pb and U-Pb+TE methods is more obvious. The spread of the data is again more visible for the U-Pb+TE method. The %2RSD from U-Pb is around 4% for Plešovice (Fig. 2a), and around 5% for FC1 (Fig. 2c). For U-Pb+TE these numbers are higher, with a %2RSD of around 5% for Plešovice (Fig. 2b), and with a bigger spread, around 6–7% for FC1 (Fig. 2d). Reported ages for Plešovice are measured using ^206^Pb/^238^U while, FC1 uses ^207^Pb/^206/^Pb ratio. The difference comes from the age of the zircons. For old zircons (over 1000 Ma) the lead ratio is used because enough time has passed for ^207^Pb to accumulate and to give a better age, whereas in the case of younger zircons, the lead ratio is not reliable.

As expected for the primary standard, SLF values were in aligntment with reference values, confirming a stable calibration throughout the analytical sessions. Overall, measurements for SLF remained within 5% of reported ages, with deviations staying within the reported error of ±16 Ma (<3%).

To further validate the U–Pb methodology, two previously dated and published samples from the Apuseni Mountains (POI-01 and HAI-01 of 24) were reanalyzed. Analyses at the British Geological Survey were conducted using a Nu Instruments Nu Plasma HR multi-collector inductively coupled plasma mass spectrometer, coupled with a New Wave Research 193ss laser ablation system. Analytical conditions included fluences between 1.5 and 2.5 J cm⁻², an ablation frequency of 5 Hz, and a beam diameter of 35 µm. Additional details regarding analytical procedures and data processing employed by the Geochronology & Tracers Facility at the British Geological Survey (GTF-BGS) are provided in the Supplementary Materials (Appendix 1, Section 1) of [24].

Concordia and weighted mean ages were calculated. For weighted mean age determinations, only analyses exhibiting ≤5% discordance between ²³⁸U/²⁰⁶Pb and ²³⁷U/²⁰⁵Pb ages were considered. Furthermore, the recommendations of [25] regarding the validity of calculated ages were followed. In some cases, reanalysis of the same crystals was not possible due to limited available surface area; consequently, minor age discrepancies may also reflect this limitation.

**Fig. 3:**
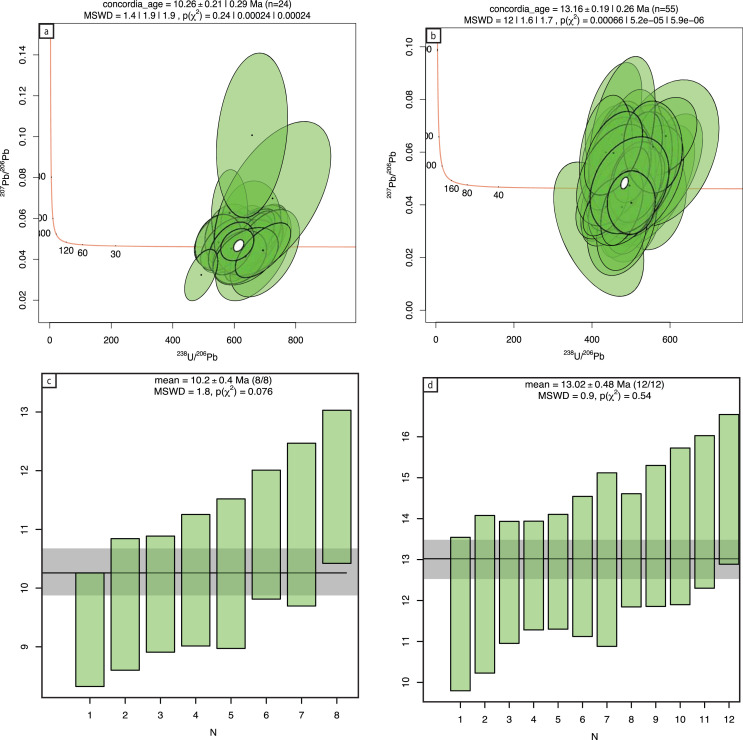
Ages calculated using the methodology from this study with 2s% error elipses, representing a 95% confidence interval. (a) Sample HAI-01 Tera-Wasserburg concordia age; (b) Sample POI-01 Tera-Wasserburg concordia age; (c) Sample HAI-01 weighted mean; (d) Sample POI-01 weighted mean.

For sample POI-01, a Concordia age of 13.16 ± 0.19 Ma was obtained, while the corresponding weighted mean age is 13.02 ± 0.48 Ma, with an MSWD of 0.9 (Fig. 3). Both ages overlap within uncertainty with the previously reported age of 13.33 ± 0.25 Ma. The second sample, HAI-01, yielded a Concordia age of 10.26 ± 0.21 Ma and a weighted mean age of 10.2 ± 0.4 Ma (MSWD = 1.8, n = 8), compared to a previously published age of 10.41 ± 0.10 Ma.

**Fig. 4:**
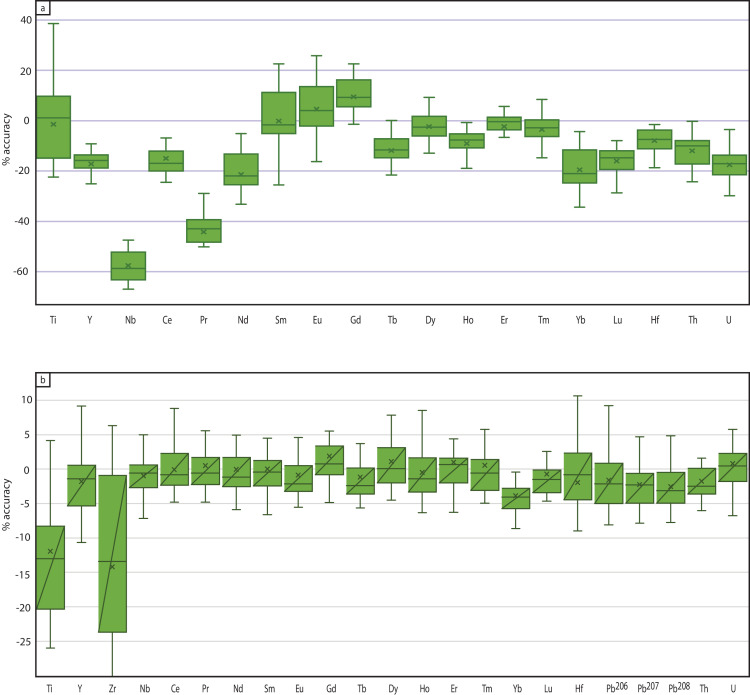
Box and whiskers plot representing variation in accuracy on SLF and NIST612 from each session for REE, represented by the difference in % of measured value with the accepted value. The horizontal line represents the average and the x represents the median. (a) SLF secondary standard; (b) NIST612 secondary standard.

The accuracy of trace elements analyses on SLF and NIST612SRM are presented in Fig. 4. For SLF, the most accurate elements that have the median accuracy better than 10% are Tb, Gd, Dy, Ho, Er, Eu, Tm, Hf, Sm and Th (in this order). Elements Ti, Y, Ce, Nd and Yb have a median accuracy better than 20%, while Lu (−21.05%) and U (−21.93%) exceed the 20% threshold. The worst performing elements with accuracies worse than 40% are Nb and Pr. Compared to SLF, the accuracies obtained for NIST612SRM are better, which is not surprising, given that NIST612SRM is a synthetic glass similar to the primary reference material NIST610SRM. The median accuracies for most elements are better than 5% except Ti and Zr which are −12.99% and −13.41 respectively.

**Fig. 5:**
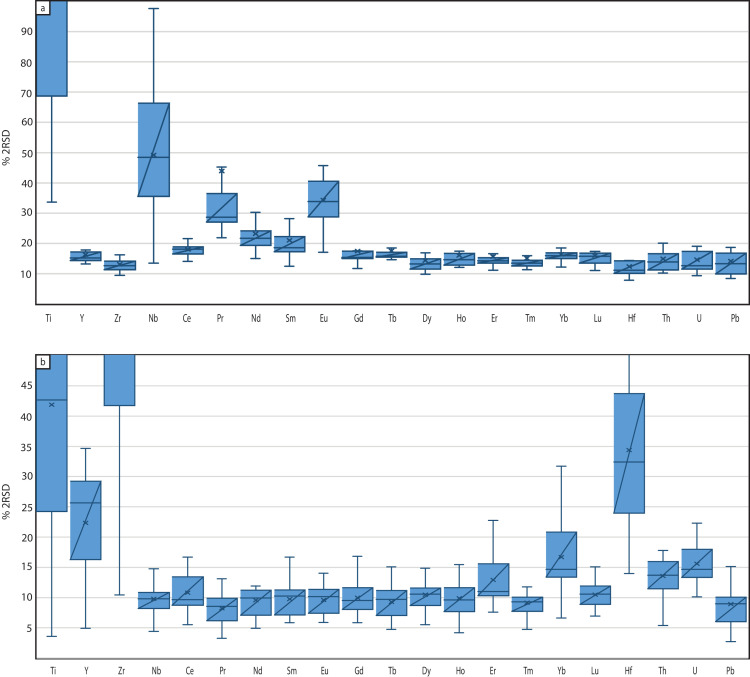
Box-and-whiskers plot representing variation in precision from each session (%2RSD) for REE. The horizontal line represents the average and the x represents the median. (a) SLF (b) NIST612.

The 2s% precision calculated for SLF and NIST612SRM is shown in Fig. 5. For SLF, most elements are within 20%, starting with Hf (11.14%), U, Zr, Dy, Tm, Th, Er, Ho, Y, Gd, Lu, Tb, Yb, Ce and ending with Sm (18.53%). There are four elements that are over the 20% threshold (Nd, 21.63%; Pr, 28.66%; Eu, 33.79%; Nb, 48.42%) and one element that is over 100% (Ti). The 2s% for NIST612SRM is under 15% for most elements. The best precision is for Pr (8.56%), followed by Tm, Gd, Ho, Ce, Tb, Nb, Nd, Eu, Sm, Dy, Lu, Er, Th, U and the last element within 20% is Yb (14.67%). There are three elements with %2RSD over the 20% limit: Hf (32.41%), Ti (42.66%) and Zr (74.81%).

**Fig. 6:**
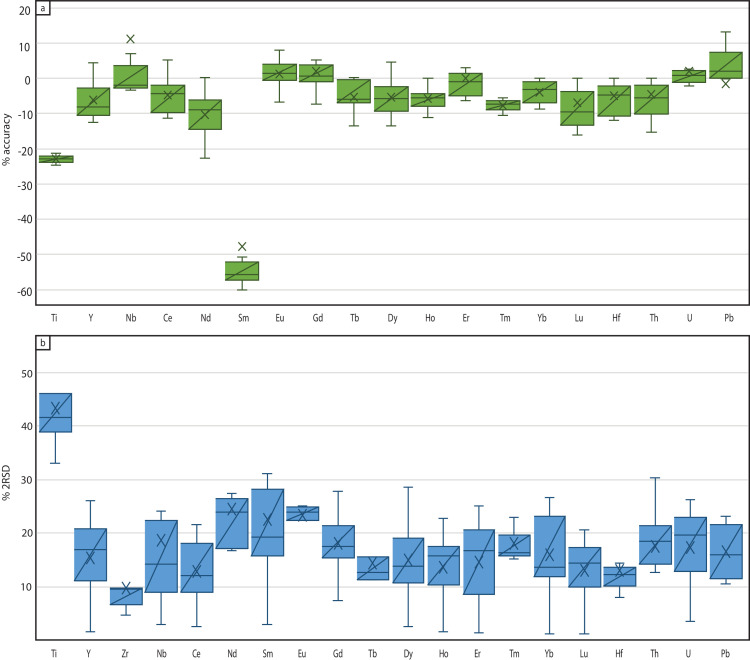
Box and whiskers plot representing variation in accuracy and precision for 91,500 as secondary standard for REE. The horizontal line represents the average and the x represents the median (a) accuracy (b) %2RSD.

The accuracy and precision for the 91,500 zircons can be seen in Fig. 6. Elements that show accuracies better than 10% accuracy are Pb, Eu, U, Gd, Nb, Er, Yb, Nd, Ce, Hf and Dy, and whereas Y, Lu and Th are between 10 and 15%. The worst performing elements are Ti (−22.89% with only 4 sessions) and Sm (−56.59%). Regarding the 2s% precision, almost all measured elements (Tb, Dy, Sm, Gd, Pb, Yb, Th, Zr, Tm, U, Ti, Y, Er, Hf, Ho, Eu, Lu, Nd) are better than 20%, with the exception of Nb (23.9%) and Ce (41.58%).

Based on over twelve months of data acquisition, the PetroChron Laboratory has demonstrated the capability to routinely perform combined zircon geochronology and geochemistry. While measuring age and geochemistry separately remains the preferred approach, simultaneous (U-Pb+TE) analysis maintains an accuracy within 5%. Precision for ages during these combined runs remains within an acceptable 3%.

The geochemical data also shows high reproducibility, with more than half of the analysed elements yielding accuracies within 10% and precision remaining within 20% across all masses. These consistent results over an extended period indicate that the method achieves the high standards of precision and accuracy required for complex geological characterization.

## Limitations

None.

## Ethics statements

This study did not involve human participants, animal experiments, or the use of personal data. Therefore, ethical approval was not required.

## CRediT authorship contribution statement

**Maria Pârlea:** Formal analysis, Data curation, Writing – original draft, Validation. **Vlad Ene:** Data curation, Writing – review & editing. **Mihai Ducea:** Supervision, Funding acquisition. **Peter Luffi:** Supervision, Methodology, Writing – review & editing. **Ciprian Stremțan:** Methodology, Writing – review & editing.

## Declaration of competing interest

The authors declare that they have no known competing financial interests or personal relationships that could have appeared to influence the work reported in this paper.

## Data Availability

Data will be made available on request.
